# Postauricular Leiomyosarcoma: A Case Report and Literature Review

**DOI:** 10.1155/2013/284275

**Published:** 2013-03-21

**Authors:** Dillip Samal, Rajeev Kumar, Saumyaranjan Mallick, Alok Thakar

**Affiliations:** ^1^ENT Department, All India Institute of Medical Sciences, Ansari Nagar, New Delhi 110029, India; ^2^Pathology Department, All India Institute of Medical Sciences, Ansari Nagar, New Delhi 110029, India

## Abstract

Leiomyosarcoma arising in the head and neck region is a rare entity. Auricular involvement by the disease is further rarer with few cases reported in the literature. Usually auricular leiomyosarcoma is a disease of middle-old age. We report a case of leiomyosarcoma of the postauricular region in a young adolescent female. Surgery along with adjuvant radiotherapy was used for complete cure. Patient is disease-free for the last eight years and is on regular yearly followup. The aim of reporting this case is to add to the scarce existing literature regarding auricular leiomyosarcoma and its long-term outcome. Also, this is the first case report in young adolescent and second only of the post auricular region.

## 1. Introduction

Leiomyosarcoma constitutes a rare group of malignant tumor of the mesenchymal origin constituting 6% of all soft tissue sarcoma. About 3% to 10% of all leiomyosarcoma are found in head and neck region [1]. Leiomyosarcoma of the ear is further rarer site in the category of head and neck region. The origin of tumor is proposed to be from smooth muscle present in walls of blood vessels and the erector pili musculature of skin [1, 2]. We report a case of leiomyosarcoma arising in the postauricular region in a 15-year-old adolescent female with long-term followup. We reviewed the Pubmed database for the term “leiomyosarcoma of ear/temporal bone” and found only 10 cases reported from 1964 to 2011. All case reports were summarized for age, sex, site of origin, treatment modality used, and follow-up period (Table 1). To the best of our knowledge, this is the first case report in the literature of postauricular leiomyosarcoma in a young adolescent with long-term followup.

## 2. Case Report

**Figure 1:**
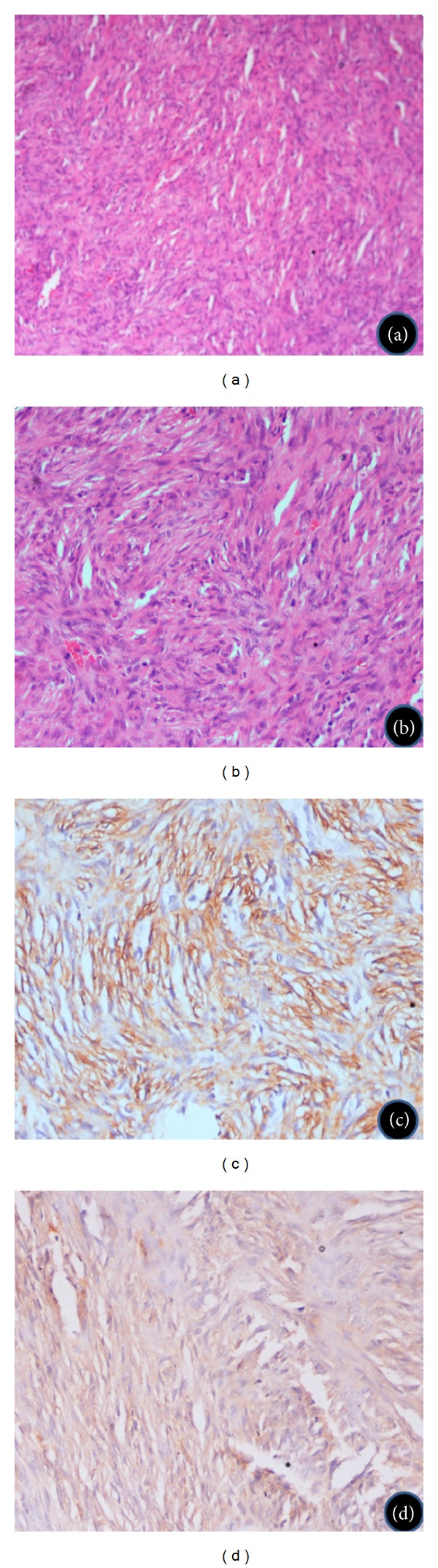
Microphotograph shows intersecting marginated groups of spindle tumor cell with elongated and blunt-ended nuclei. There is nuclear hyperchromasia and pleomorphism. ((a) H&E 100x, (b) 200x). The cells are immunopositive for smooth muscle actin ((c) IHC (SMA) 200x) and focally positive for S100 ((d) IHC (S100) 200x).

**Figure 2:**
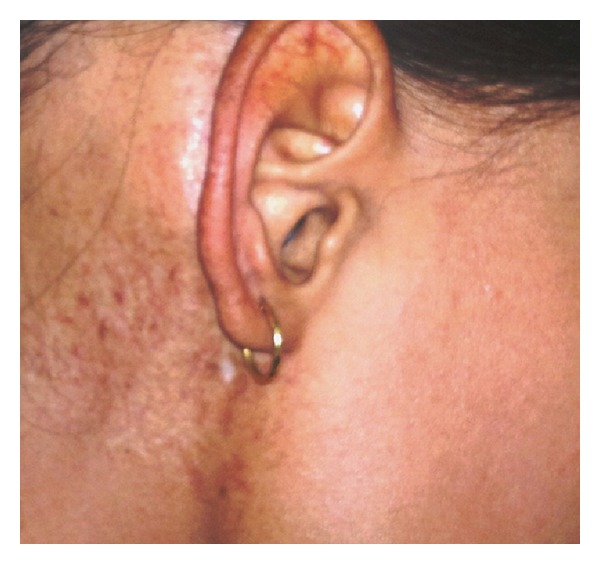
Clinical photograph of the patient showing postradiotherapy changes.

A 15-year-old female presented with 8-month history of progressive painless nodular swelling in right postauricular region. There was no history of ear discharge, ear pain, ear bleeding, or impairment of hearing. Patient had undergone local excision of swelling twice at local hospital within 1-month interval. Patient again had recurrence of swelling and for which she was referred to our centre. On examination, a 3/4 cm nontender firm nodular swelling with overlying scar mark (previous surgeries) was present in the right postauricular region. There was no palpable lymphadenopathy, and rest of the otolaryngological examination was within normal limit. Tympanic membrane and external auditory meatus was normal. Fine needle aspiration cytology (FNAC) was suggestive for features of low-grade sarcoma. Patient underwent wide local excision of the lesion with adequate margins. Postoperative histopathology showed 3.5 × 2.0 × 0.5 cm lesion with predominant spindle cell with mitotic rate of 6-7 per high power field. On further subjecting to immunohistochemistry (IHC), tumor cells showed immunoreactivity for smooth muscle antigen (SMA) and S-100 (Figures 1(a)–1(d)). Overall features were suggestive of leiomyosarcoma. However, the peripheral resected margins were found to be involved by tumor. Contrast enhanced computed tomography (CECT) revealed no bony involvement of mastoid cortex. In view of positive margins, an adjuvant treatment in the form of radical radiotherapy was given (60 Gy/30# over 6 weeks). Patient was on regular followup and disease-free for the last 8 years (Figure 2).

## 3. Discussion

Leiomyosarcoma affecting ear or temporal bone is a very rare entity. In the head and neck region, the most common site reported is sinonasal tract [1]. Rasp et al. in their review of head and neck leiomyosarcoma found scalp and other soft tissue areas to be the most common sites [3]. They are usually seen in middle to old age group (Table 1); however, in our case, patient was a young adolescent female. They may present as solitary nodule or as ulcerative lesion. Diagnosis is confirmed by histopathological evaluation of excised specimen after immunohistochemistry. On microscopy, tumor showed poorly oriented atypical spindle cells with mitoses. The number of mitoses per high power field is an important criterion and ≥2 mitoses per high power field almost certain to be malignant [1]. In our case, the specimen showed a mitotic rate of 6-7 per high power field with immunohistochemistry positive for S-100 and SMA.

The mainstay of treatment is usually wide local excision of the lesion with adequate margins. Radical resection has been suggested for more aggressive subcutaneous lesion. Although metastasis is rarely reported, if it occurs, it is generally by haematogenous route [1]. Recurrence in cases of cutaneous leiomyosarcoma is reported to be as high as 30% to 50% [1]. Radiotherapy can be used as adjuvant treatment to surgery in the setting of either aggressive or late-stage disease with poor tumor response.

## 4. Conclusion

Auricular leiomyosarcoma is a rare entity. Surgical excision with adequate margins is the treatment modality of choice. Radiotherapy can be used as an adjuvant treatment in aggressive cases.

## Figures and Tables

**Table 1:** Summary of previously reported cases of leiomyosarcoma involving ear/temporal bone.

Sl no.	Year	Author	Patient details	Site	Treatment modalities	Follow-up period
1	1964	Charlton [1]	69 yr/F	Right external auditory canal	Wide excision	NA
2	1991	Rasp et al. [3]	45 yr/M	Right EAC	Surgical removal	NA
3	1994	Zbaren and Ruchti [4]	71 yr/F	Extensive lesion of middle ear and temporal bone	Palliative chemotherapy	Died after 6 months due to disease progression
4	1995	Nilles et al. [5]	74 yr	Temporal bone	Surgical removal	NA
5	1998	Karasen [6]	68 yr/M	Left auricle	Radical resection of auricle	1 yr NAD
6	2003	Pai et al. [7]	79 yr/M	Left auricle	Partial auriculectomy	NA
7	2004	$\ddot{\text{O}}$ztürk et al. [2]	46 yr/M	Postauricular region	Wide excision with 3 cm margin	26 months NAD
8	2007	Annest et al. [8]	68 yr/M	Left Ear	Wide excision	6 months NAD
9	2010	Mehta et al. [9]	78 yr/M	Right auricle	Surgery + radiotherapy	2 yr followup, death due to metastasis
10	2011	Ursick and Linthicum [10]	60 yr/F	Left temporal bone	Autopsy histopathology	
